# The impact of endoscopist performance and patient factors on distal adenoma detection and colorectal cancer incidence

**DOI:** 10.1186/s12876-024-03125-x

**Published:** 2024-01-23

**Authors:** Sharon Power, Kate Wooldrage, Brian P. Saunders, Amanda J. Cross

**Affiliations:** 1 Cancer Screening and Prevention Research Group (CSPRG), Department of Surgery and Cancer, St Mary’s Hospital, Imperial College London, London, W2 1NY UK; 2 Department of Surgery and Cancer, Imperial College London, London, UK; 3 Department of Gastroenterology, St Mark’s Hospital and Academic Institute, London, UK

**Keywords:** Colorectal cancer, Endoscopic screening, Adenoma detection, Key performance indicators, Flexible sigmoidoscopy

## Abstract

**Background:**

High quality endoscopy is key for detecting and removing precursor lesions to colorectal cancer (CRC). Adenoma detection rates (ADRs) measure endoscopist performance. Improving other components of examinations could increase adenoma detection.

**Aims:**

To investigate how endoscopist performance at flexible sigmoidoscopy (FS) affects adenoma detection and CRC incidence.

**Methods:**

Among 34,139 participants receiving FS screening by the main endoscopist at one of 13 centres in the UK FS Screening Trial, median follow-up was 17 years. Factors examined included family history of CRC, bowel preparation quality, insertion and withdrawal time, bowel segment reached, patient pain and ADR. Odds ratios (OR) for distal adenoma detection were estimated by logistic regression. Hazard ratios (HR) for distal CRC incidence were estimated by Cox regression.

**Results:**

At screening, 4,104 participants had distal adenomas detected and 168 participants developed distal CRC during follow-up. In multivariable models, a family history of CRC (yes vs. no: OR 1.40, 95%CI 1.21–1.62), good or adequate bowel preparation quality (vs. excellent: OR 0.84, 95%CI 0.74–0.95; OR 0.56, 95%CI 0.49–0.65, respectively) and longer insertion and withdrawal times (≥ 4.00 vs. < 2.00 min: OR 1.96, 95%CI 1.68–2.29; OR 32.79, 95%CI 28.22–38.11, respectively) were associated with adenoma detection. Being screened by endoscopists with low or intermediate ADRs, compared to high ADRs, was positively associated with CRC incidence (multivariable: HR 4.71, 95%CI 2.65–8.38; HR 2.16, 95%CI 1.22–3.81, respectively).

**Conclusions:**

Bowel preparation quality and longer insertion and withdrawal time are key for improving distal adenoma detection. Higher ADRs were associated with a lower risk of distal CRC.

**Supplementary Information:**

The online version contains supplementary material available at 10.1186/s12876-024-03125-x.

## Introduction

Colorectal cancer (CRC) is the fourth most common cancer with over 42,000 cases diagnosed in the UK annually [1]. Effective screening for CRC enables the removal of precursor lesions, preventing CRC, and the detection of CRC at an earlier stage, significantly improving patient outcomes [2, 3].

Flexible sigmoidoscopy (FS) involves inserting a thin tube into the rectum to visualise ~ 60 cm of the distal colorectum [4]. FS screening reduces CRC incidence and mortality [5–8]; in the UK Flexible Sigmoidoscopy Screening Trial (UKFSST), CRC incidence and mortality was reduced by 35% and 41%, respectively, in those screened compared to controls [9].

Endoscopic examination accuracy is dependent on endoscopist skill and experience, with higher quality exams associated with better patient outcomes [10]. Adenoma detection rates (ADRs) are used to assess endoscopist performance [11]. Low ADRs are associated with higher rates of interval CRCs [12] and post-colonoscopy CRC mortality [13]. Large variability exists in ADRs between endoscopists [14–16], with quality of bowel preparation [17, 18], depth of endoscope insertion, segment of bowel reached and withdrawal time all related to ADRs [14]. Higher quality withdrawal techniques are associated with lower miss rates for adenomas [19].

The Joint Advisory Group on gastrointestinal endoscopy, the British Society of Gastroenterology and the Association of Coloproctology of Great Britain and Ireland have developed key performance indicators (KPIs) for endoscopy, which include ADRs, bowel preparation quality, withdrawal time, comfort and completeness of examination [20]. These KPIs are accompanied by quality assurance measures, which provide minimal standards and aspirational targets for endoscopists [20]. However, there is a lack of data on KPIs and long-term outcomes. The UKFSST offers the opportunity to examine KPIs in relation to adenoma detection and distal CRC incidence.

## Methods

### Study design

**Fig. 1:**
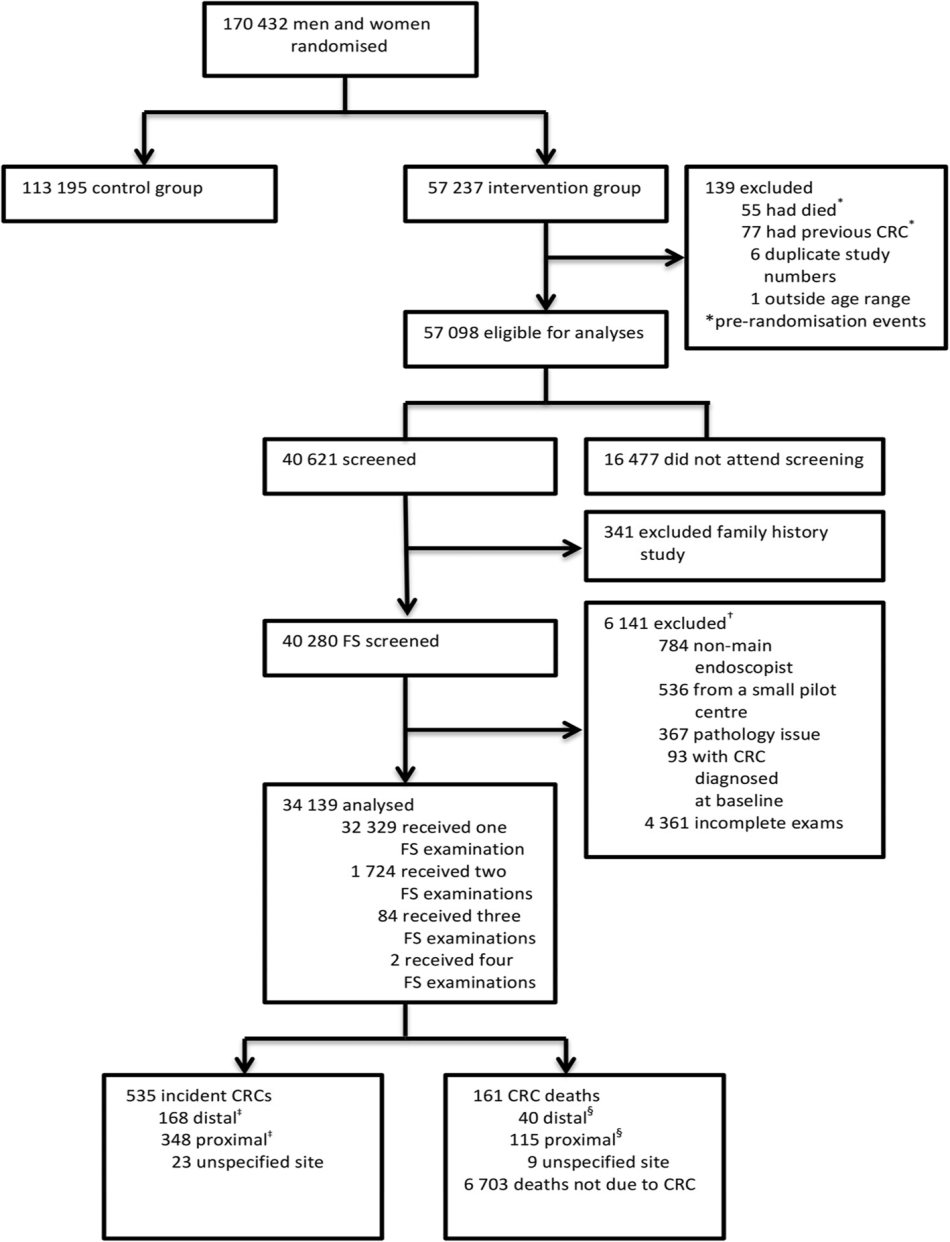
Study Profile. ^†^784 patients whose FS screening was performed by an endoscopist other than the main endoscopist at that centre, 536 patients screened at one pilot centre that had far fewer participants than the other centres and where there were two main endoscopists rather than one, 367 patients who were screened within the first two months at one centre where the pathologist was found to be over-diagnosing adenomas, 93 participants with CRC diagnosed at baseline and 4,361 participants whose exam was classed as incomplete by the endoscopist. ^‡^Four patients had incident CRC diagnosed at both sub-sites. ^§^Three patients had CRC as the underlying cause of death but the sub-site specific cause could not be determined as CRC was diagnosed at both sub-sites

Between November 1994 and March 1999, the UKFSST recruited men and women aged 55–64 years from general practices serving 14 UK hospitals; details reported previously [9]. Adenoma incidence increases after the age of 50 years but levels out before 60 years [15, 21]; screening around 60 years of age offered the optimum opportunity to detect adenomas [21]. Participants were excluded if they were unable to provide consent; had a history of CRC, adenomas or inflammatory bowel disease; had severe/terminal disease, life expectancy of < 5 years, or a sigmoidoscopy/colonoscopy within the previous 3 years. Eligible individuals were randomised to either the intervention (*n* = 57,237, invitation to once-only FS screening), or control arm (*n* = 113,195, no screening and no further contact) (Fig. 1).

We excluded those who died or were diagnosed with CRC pre-randomisation, those in a family history study receiving colonoscopy screening, those screened by an endoscopist other than the main endoscopist at each centre, those in the pilot centre, those screened within the first two months at one centre where the pathologist was over-diagnosing adenomas, those diagnosed with CRC at baseline and those with incomplete exams (Fig. 1). Of the 34,139 participants remaining, 1,810 received multiple FS examinations (89% repeated due to poor bowel preparation quality; Supplementary Table 1). Only one exam per participant was included in the analysis; if FS was repeated due to poor bowel preparation, the last complete exam was included, but if FS was repeated for other reasons, the earliest complete exam was used. If any exams had polyps detected, these rules were applied within exams with polyps only.

Endoscopists were registrar-level gastroenterologists/surgeons with 3–8 years of experience post-basic medical qualification and must have performed a minimum of 50 supervised and 100 unsupervised endoscopies [15]. Participants were to administer a single phosphate enema (Fletchers’ phosphate enema; Forest Laboratories UK Ltd., Bexley, Kent), one hour before leaving home for their examination [15]. Sedation was not routinely used during FS examination [15]. All endoscopists were to advance the scope (60 cm Olympus video-endoscope (CF-200S)) as far as possible without causing undue discomfort (normally to the sigmoid colon/descending colon junction) and to remove polyps < 10 mm, leaving intact polyps < 3 mm deemed to be hyperplastic in the distal 4 cm of the rectum [15]. Follow-up colonoscopy was arranged for participants at high risk (≥ 3 adenomas, a polyp ≥ 10 mm, an adenoma with villous/tubulovillous histology, or high-grade dysplasia, malignant disease, or $\ge$ 20 hyperplastic polyps above the distal rectum) [15].

### Exposures

We examined endoscopist-reported variables of bowel preparation quality (Supplementary Table 2), time to maximum point of insertion, withdrawal time from maximum point of insertion, and segment of bowel reached. A pre-examination questionnaire assessed family history of CRC in first-degree relatives and a post-examination questionnaire assessed the level of pain experienced (none, mild, quite a lot, severe) during FS.

UKFSST endoscopists were previously ranked by their ADR (estimated as the proportion of participants that had $\ge$ 1 distal adenoma detected) into high-, intermediate-, or low-detectors, with corresponding ADRs of 15%, 12% and 9%, respectively [15]; these groups were used in this analysis. The order in which participants were screened revealed a learning effect for the endoscopists’ ADR [15]; thus, we created a variable that grouped participants according to the order of examination occurrence: the first 500 participants examined by each endoscopist and those examined later.

### Outcome ascertainment

Information on date, site and morphology of cancers and date of emigrations and deaths were collected from National cancer registries, the National Health Service (NHS) Central Register, National Services Scotland, NHS Digital and the Office for National Statistics.

Primary outcomes were distal adenomas and distal CRC incidence. Distal adenomas included adenomas detected at FS or any distal adenoma detected at follow-up colonoscopy (endoscopists were to leave polyps ≥ 10 mm for removal at colonoscopy). Distal CRCs, defined by the International Classification of Diseases 10^th^ revision (ICD-10) and ICD for Oncology 2^nd^ edition [22], included sites C18.7, C19 and C20 (rectum and sigmoid colon) and morphologies for invasive adenocarcinomas and carcinomas not otherwise specified for cancers diagnosed on clinical grounds only. The earliest distal CRC diagnosed per patient was included and follow-up time was not censored at diagnosis of proximal or unspecified site CRC.

### Statistical analysis

Univariable and multivariable logistic regression was used to estimate odds ratios (OR) and 95% confidence intervals (CIs) for associations with distal adenoma detection. For distal CRC incidence, Cox models were used to estimate hazard ratios (HR) and 95% CIs. Time-at-risk started from baseline FS examination and was censored at emigration, death or the end of 2014. Non-proportionality was assessed using the Schoenfeld test; no violations were identified.

Initial univariable analyses included everyone with complete data on each variable, referred to as “full dataset” analyses. Multivariable analyses required data for all variables in the model, referred to as “complete-case” analyses; see Tables 1 and 2 for details. Insertion and withdrawal times were missing in ~ 40% of participants as this was not recorded until partway through the trial. Further sensitivity analyses were conducted excluding participants with multiple FS examinations.

**Table 1:** Detection of any distal adenoma by patient factors and endoscopist variables

	**All eligible participants: Full dataset analysis (*n* = 34 139)**				**Participants with complete data on all variables: Complete-case analysis (*n* = 19 333)**^**a**^					
	**n (%)**^b^	**Participants with ≥ 1 adenoma detected at baseline n (%)**	**Univariable OR (95%CI)**	***p*-value**	**n (%)**^b^	**Participants with ≥ 1 adenoma detected at baseline n (%)**	**Univariable OR (95%CI)**	***p*-value**	**Multivariable OR (95%CI)**^c^	***p*-value**
**Total**	**34 139 (100)**	**4 104 (12.0)**			**19 333 (100)**	**2 415 (12.5)**				
**Age (IQR), years**	**60.3 (57.9–62.8)**	**4 104 (12.0)**	1.03 (1.02–1.04)	< 0.001	60.5 (58.0–62.9)	2 415 (12.5)	1.03 (1.01–1.05)	< 0.001	1.03 (1.01–1.04)	0.002
**Sex**	**34 139 (100)**	**4 104 (12.0)**		< 0.001				< 0.001		< 0.001
Male	18 127 (53.1)	2 817 (15.5)	1		10 315 (53.4)	1 665 (16.1)	1		1	
Female	16 012 (46.9)	1 287 (8.0)	0.48 (0.44–0.51)		9 018 (46.6)	750 (8.3)	0.47 (0.43–0.52)		0.62 (0.56–0.69)	
**Family history of CRC**	**32 356 (94.8)**	**3 926 (12.1)**		< 0.001				< 0.001		< 0.001
No	28 663 (88.6)	3 381 (11.8)	1		17 123 (88.6)	2 077 (12.1)	1		1	
Yes	3 693 (11.4)	545 (14.8)	1.29 (1.17–1.43)		2 210 (11.4)	338 (15.3)	1.31 (1.15–1.48)		1.40 (1.21–1.62)	
**Centre**	**34 139 (100)**	**4 104 (12.0)**		< 0.001				< 0.001		< 0.001
1	2 413 (7.1)	207 (8.6)	1		1 607 (8.3)	139 (8.6)	1		1	
2^d^	3 438 (10.1)	302 (8.8)	1.03 (0.85–1.23)		-	-	-		-	
3	2 674 (7.8)	249 (9.3)	1.09 (0.90–1.33)		1 452 (7.5)	135 (9.3)	1.08 (0.84–1.39)		0.91 (0.67–1.23)	
4	2 131 (6.2)	209 (9.8)	1.16 (0.95–1.42)		1 452 (7.5)	146 (10.1)	1.18 (0.93–1.51)		1.55 (1.14–2.09)	
5	2 466 (7.2)	271 (11.0)	1.32 (1.09–1.59)		1 844 (9.5)	189 (10.2)	1.21 (0.96–1.52)		0.41 (0.31–0.54)	
6	2 733 (8.0)	306 (11.2)	1.34 (1.12–1.62)		1 579 (8.2)	163 (10.3)	1.22 (0.96–1.54)		1.23 (0.92–1.65)	
7	2 516 (7.4)	282 (11.2)	1.35 (1.11–1.62)		1 741 (9.0)	205 (11.8)	1.41 (1.12–1.77)		1.19 (0.89–1.58)	
8	2 839 (8.3)	362 (12.8)	1.56 (1.30–1.86)		1 486 (7.7)	190 (12.8)	1.55 (1.23–1.95)		0.77 (0.58–1.03)	
9	2 493 (7.3)	349 (14.0)	1.73 (1.45–2.08)		1 617 (8.4)	236 (14.6)	1.80 (1.45–2.25)		1.39 (1.06–1.83)	
10	2 532 (7.4)	370 (14.6)	1.82 (1.52–2.18)		1 578 (8.2)	215 (13.6)	1.67 (1.33–2.09)		0.57 (0.43–0.76)	
11	2 324 (6.8)	347 (14.9)	1.87 (1.56–2.25)		1 202 (6.2)	185 (15.4)	1.92 (1.52–2.43)		0.65 (0.49–0.86)	
12	2 799 (8.2)	421 (15.0)	1.89 (1.58–2.25)		2 214 (11.5)	348 (15.7)	1.97 (1.60–2.43)		1.11 (0.86–1.44)	
13	2 781 (8.1)	429 (15.4)	1.94 (1.63–2.32)		1 561 (8.1)	264 (16.9)	2.15 (1.73–2.67)		0.71 (0.54–0.92)	
**Bowel preparation quality**	**33 609 (98.4)**	**3 925 (11.7)**		0.05				0.001		< 0.001
Excellent	14 573 (43.4)	1 690 (11.6)	1		7 819 (40.4)	924 (11.8)	1		1	
Good	11 692 (34.8)	1 431 (12.2)	1.06 (0.99–1.15)		6 922 (35.8)	918 (13.3)	1.14 (1.03–1.26)		0.84 (0.74–0.95)	
Adequate	7 060 (21.0)	769 (10.9)	0.93 (0.85–1.02)		4 553 (23.6)	561 (12.3)	1.05 (0.94–1.17)		0.56 (0.49–0.65)	
Poor	284 (0.8)	35 (12.3)	1.07 (0.75–1.53)		39 (0.2)	12 (30.8)	3.32 (1.67–6.57)		2.88 (1.25–6.60)	
**Insertion time**	**20 371 (59.7)**	**2 630 (12.9)**		< 0.001				< 0.001		< 0.001
< 2.00 mins	7 357 (36.1)	795 (10.8)	1		7 014 (36.3)	727 (10.4)	1		1	
2.00–2.59 mins	6 198 (30.4)	759 (12.2)	1.15 (1.04–1.28)		5 894 (30.5)	702 (11.9)	1.17 (1.05–1.31)		1.18 (1.04–1.35)	
3.00–3.59 mins	3 412 (16.7)	466 (13.7)	1.31 (1.16–1.48)		3 221 (16.7)	424 (13.2)	1.31 (1.15–1.49)		1.37 (1.18–1.61)	
$\ge$ 4.00 mins	3 404 (16.7)	610 (17.9)	1.80 (1.61–2.02)		3 204 (16.6)	562 (17.5)	1.84 (1.63–2.07)		1.96 (1.68–2.29)	
**Withdrawal time**	**20 326 (59.5)**	**2 621 (12.9)**		< 0.001				< 0.001		< 0.001
< 2.00 mins	10 625 (52.3)	295 (2.8)	1		10 204 (52.8)	262 (2.6)	1		1	
2.00–2.59 mins	3 672 (18.1)	295 (8.0)	3.06 (2.59–3.61)		3 479 (18.0)	266 (7.6)	3.14 (2.64–3.74)		3.85 (3.21–4.62)	
3.00–3.59 mins	1 837 (9.0)	311 (16.9)	7.14 (6.03–8.44)		1 740 (9.0)	289 (16.6)	7.56 (6.34–9.01)		9.47 (7.88–11.38)	
$\ge$ 4.00 mins	4 192 (20.6)	1 720 (41.0)	24.36 (21.37–27.78)		3 910 (20.2)	1 598 (40.9)	26.23 (22.84–30.12)		32.79 (28.22–38.11)	
**Segment reached**	**34 075 (99.8)**	**4 098 (12.0)**		< 0.001				< 0.001		0.79
RM/RS/SC	128 (0.4)	16 (12.5)	1.43 (0.84–2.44)		53 (0.3)	8 (15.1)	1.63 (0.76–3.48)		1.12 (0.46–2.69)	
SD	7 510 (22.0)	680 (9.1)	1		4 447 (23.0)	437 (9.8)	1		1	
DC	21 327 (62.6)	2 660 (12.5)	1.43 (1.31–1.56)		11 840 (61.2)	1 519 (12.8)	1.35 (1.21–1.51)		1.00 (0.87–1.14)	
SF	3 462 (10.2)	491 (14.2)	1.66 (1.47–1.88)		2 062 (10.7)	310 (15.0)	1.62 (1.39–1.90)		1.12 (0.92–1.38)	
TC/HF/AC/CM/TI	1 648 (4.8)	251 (15.2)	1.80 (1.54–2.11)		931 (4.8)	141 (15.1)	1.64 (1.33–2.01)		1.01 (0.79–1.28)	
**Patient pain**	**33323 (97.6)**	**3 989 (12.0)**		0.66				0.003		0.48
None	9 563 (28.7)	1 164 (12.2)	1		4 937 (25.5)	685 (13.9)	1		1	
Mild	17 859 (53.6)	2 139 (12.0)	0.98 (0.91–1.06)		10 561 (54.6)	1 292 (12.2)	0.87 (0.78–0.96)		0.92 (0.82–1.03)	
Quite a lot	5 224 (15.7)	613 (11.7)	0.96 (0.86–1.06)		3 378 (17.5)	392 (11.6)	0.81 (0.71–0.93)		0.92 (0.78–1.07)	
Severe	677 (2.0)	73 (10.8)	0.87 (0.68–1.12)		457 (2.4)	46 (10.1)	0.69 (0.51–0.95)		0.86 (0.60–1.25)	
**FS occurrence**^e^	**34 139 (100)**	**4 104 (12.0)**		< 0.001				-		-
First group 500	5 410 (15.8)	526 (9.7)	1		-	-	-		-	
Later groups 500	28 729 (84.2)	3 578 (12.5)	1.32 (1.20–1.45)		-	-	-		-	

*Abbreviations*: *AC* ascending colon, *CI* confidence interval, *CM* caecum, *DC* descending colon, *FS* flexible sigmoidoscopy, *HF* hepatic flexure, *Mins* minutes, *OR* odds ratio, *RM* rectum, *RS* recto sigmoid, *SC* sigmoid colon, *SD* sigmoid descending, *SF* splenic flexure, *TC* transverse colon, *TI* terminal ileum

*P*-values were calculated with the likelihood ratio test

^a^1 783 missing values on family history of CRC; 530 missing values on bowel preparation quality; 13 768 missing values on insertion time; 13 813 missing values on withdrawal time; 64 missing values on segment reached; 816 missing values on patient-reported pain (these values are not mutually exclusive)

^b^All n and percentage except the entry for age, which is median and interquartile range

^c^Multivariable model includes age, sex, family history of CRC, centre, bowel preparation quality, insertion time, withdrawal time, segment reached and patient-reported pain

^d^Centre 2 was omitted from the complete-case analyses due to a lack of recorded information for insertion or withdrawal times, as this information was not required until partway through the trial at which time centre 2 had already completed recruitment

^e^Order of occurrence of FS examination was omitted from the complete-case analyses due to a lack of recorded information for insertion and withdrawal times for the category ‘first group 500’; this information was not required until partway through the trial at which time each endoscopist had already completed 500 examinations

**Table 2:** Long-term distal colorectal cancer incidence by patient factors and endoscopist variables

	**All eligible participants: Full dataset analysis (*n* = 34 139)**					**Participants with complete data on all variables: Complete-case analysis (*n* = 19 294)**^a,b^						
	**n (%)**^c^	**Number of distal CRCs, n**	**Incidence rate per 100,000 person-years (95% CI)**	**Univariable HR (95%CI)**	***p*-value**	**n (%)**^c^	**Number of distal CRCs, n**	**Incidence rate per 100,000 person-years (95% CI)**	**Univariable HR (95%CI)**	***p*-value**	**Multivariable HR (95%CI)**^d^	***p*-value**
**Total**	**34 139 (100)**	**168**	31.4 (27.0–36.5)			**19 294 (100)**	**91**	30.8 (25.1–37.9)				
**Age (IQR), years**	**60.3 (57.9–62.8)**	**168**	-	1.03 (0.98–1.09)	0.23	**60.5 (58.0–62.9)**	91	-	1.00 (0.93–1.08)	0.98	1.00 (0.93–1.08)	0.96
**Sex**	**34 139 (100)**	**168**			0.003					0.06		0.12
Male	18 127 (53.1)	106	38.1 (31.5–46.1)	1		10 294 (53.4)	56	36.2 (27.9–47.1)	1		1	
Female	16 012 (46.9)	62	24.1 (18.8–31.0)	0.62 (0.45–0.85)		9 000 (46.7)	35	24.9 (17.9–34.7)	0.67 (0.44–1.03)		0.71 (0.45–1.09)	
**Family history of CRC**	**32 356 (94.8)**	**156**			0.026					0.23		0.24
No	28 663 (88.6)	129	28.8 (24.2–34.2)	1		17 087 (88.6)	77	29.4 (23.5–36.8)	1		1	
Yes	3 693 (11.4)	27	47.1 (32.3–68.7)	1.65 (1.09–2.50)		2 207 (11.4)	14	42.0 (24.9–70.9)	1.44 (0.81–2.54)		1.43 (0.81–2.52)	
**Bowel preparation quality**	**33 609 (98.4)**	**164**			0.19					0.45		0.16
Excellent	14 573 (43.4)	62	26.8 (20.9–34.4)	1		7 819 (40.5)	33	27.4 (19.5–38.5)	1		1	
Good	11 692 (34.8)	56	30.7 (23.6–39.9)	1.15 (0.80–1.65)		6 922 (35.9)	32	30.3 (21.4–42.9)	1.11 (0.69–1.81)		1.24 (0.76–2.03)	
Adequate	7 060 (21.0)	44	40.5 (30.1–54.4)	1.53 (1.04–2.25)		4 553 (23.6)	26	37.8 (25.7–55.5)	1.40 (0.83–2.33)		1.72 (0.99–2.97)	
Poor^b^	284 (0.8)	2	44.8 (11.2–178.9)	1.65 (0.40–6.74)		-	-	-	-		-	
**Insertion time**	**20 371 (59.7)**	**93**			0.53					0.42		0.20
< 2.00 mins	7 357 (36.1)	31	27.6 (19.4–39.2)	1		7 006 (36.3)	30	28.0 (19.6–40.1)	1		1	
2.00–2.59 mins	6 198 (30.4)	27	28.5 (19.5–41.6)	1.03 (0.62–1.73)		5 885 (30.5)	27	30.0 (20.6–43.7)	1.07 (0.63–1.79)		1.09 (0.64–1.84)	
3.00–3.59 mins	3 412 (16.7)	14	26.7 (15.8–45.1)	0.96 (0.51–1.81)		3 216 (16.7)	13	26.3 (15.3–45.3)	0.93 (0.49–1.79)		0.95 (0.49–1.86)	
$\ge$ 4.00 mins	3 404 (16.7)	21	40.6 (26.5–62.3)	1.47 (0.84–2.55)		3 187 (16.5)	21	43.3 (28.2–66.4)	1.54 (0.88–2.69)		1.81 (1.00–3.27)	
**Withdrawal time**	**20 326 (59.5)**	**93**			0.24					0.27		0.09
< 2.00 mins	10 625 (52.3)	42	25.8 (19.1–34.9)	1		10 190 (52.8)	42	26.9 (19.9–36.4)	1		1	
2.00–2.59 mins	3 672 (18.1)	15	26.5 (16.0–44.0)	1.02 (0.56–1.83)		3 469 (18.0)	15	28.1 (16.9–46.6)	1.03 (0.57–1.86)		1.00 (0.55–1.82)	
3.00–3.59 mins	1 837 (9.0)	9	32.0 (16.7–61.5)	1.23 (0.60–2.52)		1 738 (9.0)	8	30.1 (15.1–60.2)	1.11 (0.52–2.36)		1.19 (0.55–2.57)	
$\ge$ 4.00 mins	4 192 (20.6)	27	42.7 (29.3–62.3)	1.64 (1.01–2.67)		3 897 (20.2)	26	44.2 (30.1–64.9)	1.63 (1.00–2.66)		1.93 (1.14–3.24)	
**Segment reached**	**34 075 (99.8)**	**167**			0.50					0.42		0.45
RM/RS/SC/SD	7 638 (22.4)	36	30.0 (21.7–41.7)	1		4 484 (23.2)	18	26.2 (16.5–41.6)	1		1	
DC	21 327 (62.6)	111	33.2 (27.6–40.0)	1.11 (0.76–1.61)		11 822 (61.3)	62	34.2 (26.7–43.9)	1.30 (0.77–2.20)		0.99 (0.57–1.71)	
SF/TC/HF/AC/CM/TI	5 110 (15.0)	20	25.1 (16.2–39.0)	0.84 (0.49–1.46)		2 988 (15.5)	11	24.3 (13.5–43.9)	0.93 (0.44–1.98)		0.67 (0.31–1.44)	
**Patient pain**	**33 323 (97.6)**	**163**			0.25					0.49		0.81
None	9 563 (28.7)	55	36.7 (28.2–47.9)	1		4 929 (25.5)	28	37.4 (25.8–54.1)	1		1	
Mild	17 859 (53.6)	85	30.4 (24.6–37.6)	0.83 (0.59–1.16)		10 541 (54.6)	47	29.1 (21.9–38.8)	0.78 (0.49–1.24)		0.87 (0.54–1.40)	
Quite a lot/severe^e^	5 901 (17.7)	23	24.8 (16.5–37.3)	0.68 (0.42–1.10)		3 824 (19.8)	16	27.2 (16.7–44.4)	0.73 (0.39–1.34)		0.83 (0.44–1.58)	
**FS occurrence**^f^	**34 139 (100)**	**168**			0.18	-	-		-			-
First group 500	5 410 (15.8)	35	39.8 (28.5–55.4)	1		-	-	-	-	-	-	
Later groups 500	28 729 (84.2)	133	29.8 (25.1–35.3)	0.77 (0.53–1.12)		-	-	-	-	-	-	
**Endoscopist’s ADR ranking group**	**34 139 (100)**	**168**			0.001					< 0.001		< 0.001
High	12 929 (37.9)	44	21.8 (16.2–29.3)	1		8 161 (42.3)	22	17.5 (11.5–26.6)	1		1	
Intermediate	10 554 (30.9)	51	30.9 (23.5–40.7)	1.42 (0.95–2.13)		6 626 (34.3)	32	31.5 (22.3–44.5)	1.81 (1.05–3.12)		2.16 (1.22–3.81)	
Low	10 656 (31.2)	73	43.5 (34.6–54.7)	1.99 (1.37–2.90)		4 507 (23.4)	37	54.8 (39.7–75.6)	3.24 (1.91–5.49)		4.71 (2.65–8.38)	

*Abbreviations*: *AC* ascending colon, *ADR* adenoma detection rate, *CI* confidence interval, *CM* caecum, *CRC* colorectal cancer, *DC* descending colon, *FS* flexible sigmoidoscopy, *HF* hepatic flexure, *HR* hazard ratio, *Mins* minutes, *RM* rectum, *RS* recto sigmoid, *SC* sigmoid colon, *SD* sigmoid descending, *SF* splenic flexure, *TC* transverse colon, *TI* terminal ileum

*P*-values were calculated with the likelihood ratio test

^a^1 783 missing values on family history; 530 missing values on bowel preparation quality; 13 768 missing values on insertion time; 13 813 missing values on withdrawal time; 64 missing values on segment reached; 816 missing values on patient-reported pain (these values are not mutually exclusive)

^b^Participants with the ‘poor’ category of bowel preparation quality (*n* = 39) were excluded from the complete-case analyses due to a lack of cases

^c^All n and percentage except the entry for age, which is median and interquartile range

^d^Multivariable model includes age, sex, family history of CRC, bowel preparation quality, insertion time, withdrawal time, segment reached, patient-reported pain and endoscopist’s ADR ranking group

^e^Participants with the ‘severe’ category of patient-reported pain were combined with the category ‘quite a lot’ due to a lack of cases

^f^Order of occurrence of FS examination was omitted from the complete-case analyses due to a lack of recorded information for the variables of insertion and withdrawal time for the category ‘first group 500’; this information was not required until partway through the trial at which time each endoscopist had already completed 500 examinations

Multivariable models were constructed based on *a-priori* plans using previous research [14, 23] and included: age, sex, family history of CRC, bowel preparation quality, insertion time, withdrawal time, segment of bowel reached and patient-reported pain. The multivariable model for distal adenoma detection also included centre while that for distal CRC incidence included endoscopist ADR group and centre was omitted due to collinearity with ADR group. Kaplan–Meier estimates show time to distal CRC diagnosis.

Negative examinations were those with no findings in the colorectum (no lesions detected, no biopsies performed). Among those with negative examinations, we examined variation in KPIs, the associations between insertion time and pain and segment reached, and the associations between bowel preparation quality and pain and reaching the splenic flexure (SF). To examine if KPIs were associated with complexity of findings at FS, we investigated associations with the outcome of detection of multiple adenomas and/or any advanced adenoma (defined as adenomas $\ge$ 10 mm, with high-grade dysplasia, or with villous/tubulovillous histology).

Analyses were performed using STATA/IC V.13.1 (StataCorp LP, 2013; Stata Statistical Software: Release 13; Texas, USA). Two-sided *p*-values < 0.05 were considered statistically significant. Ethical approval was obtained from local research ethics review committees for each centre (Multicentre Research Ethics Committee reference: 03/01/22). Trial registration: ISRCTN28352761. All individuals who underwent FS provided written informed consent prior to examination. The Patient Information Advisory Group (now Confidentiality Advisory Group) granted permission to obtain and process patient data (PIAG 4–07(j)/2002). All methods were carried out according to the relevant guidelines.

## Results

The median age at FS was 60 years, 53% of participants were males and 11% had ≥ 1 first degree relative with CRC (Table 1). Bowel preparation quality was excellent for 43%. Median insertion and withdrawal times were 2.4 (IQR 1.7–3.4) and 1.9 (IQR 1.2–3.4) minutes, respectively. Most examinations reached the descending colon or further (78%) and 29% of participants reported feeling no pain during the examination (Table 1).

Variables were examined by centre, synonymous with endoscopist, among the 70% of participants with negative examinations (Supplementary Table 3). ‘Excellent’ bowel preparation quality varied between 9.6% (centre 9) and 68.2% (centre 4). Median insertion time varied from 1.45 (IQR 1.03–2.07; centre 4) to 3.88 (IQR, 2.92–5.50; centre 10) minutes and median withdrawal time varied from 0.88 (IQR 0.65–1.27; centre 4) to 2.38 (IQR 1.90–3.06; centre 5) minutes. Examinations reaching the descending colon varied between 27.7% (centre 13) and 84.1% (centre 8) and participants reporting severe pain varied between 0.2% (centre 4) and 4.1% (centre 8). Despite these differences between centres, there were no clear associations between these factors and endoscopist ADR when examining by ascending order of ADR (Supplementary Table 3).

Among negative examinations, the proportion of participants reporting quite a lot/severe pain tended to decrease with further segment reached (*p*-trends < 0.001). Females were more likely to report quite a lot/severe pain than males (15.5% vs. 8.0%, respectively, among exams reaching a maximum of the SF) and to have a longer time to maximum insertion for each section of the bowel reached (SF: median 2.37 min (IQR 1.75–3.45) vs. 2.14 min (IQR 1.58–2.90)) (Supplementary Table 4).

In complete-case analyses, 3,349 (14.4%) negative examinations reached at least the SF. Females were less likely to have an examination reaching the SF (10.9%) than males (18.2%) (multivariable: OR 0.57, 95%CI 0.53–0.62). Among those with negative exams, the odds of reaching the SF were 75% lower with ‘poor’ bowel preparation compared to ‘excellent’ (multivariable: OR 0.25 95%CI 0.13–0.50) and 47% lower with the reporting of severe pain compared to no pain (multivariable: OR 0.53 95%CI 0.38–0.73) (Supplementary Table 5).

### Distal adenoma detection

There were 4,104 (12.0%) participants with ≥ 1 distal adenoma detected (Table 1). In all models, there were increased odds of distal adenoma detection with increasing age (multivariable: OR 1.03, 95%CI 1.01–1.04) (Table 1), with a family history of CRC, compared to without (multivariable: OR 1.40, 95%CI 1.21–1.62), and decreased odds in females compared to males (multivariable: OR 0.62, 95%CI 0.56–0.69).

Although there was no association in the full dataset, in complete-case models there were increased odds of distal adenoma detection for those with ‘poor’ bowel preparation compared to ‘excellent’ (multivariable: OR 2.88, 95%CI 1.25–6.60; Table 1), and lower odds for those with ‘good’ (multivariable: OR 0.84, 95%CI 0.74–0.95) or ‘adequate’ bowel preparation (multivariable: OR 0.56, 95%CI 0.49–0.65).

In all models, increasing insertion and withdrawal times were associated with distal adenoma detection (multivariable: OR ≥ 4.00 vs. < 2.00 min: 1.96, 95%CI 1.68–2.29; 32.79, 95%CI 28.22–38.11, respectively). In comparison to reaching the sigmoid/descending junction, reaching more proximally was associated with higher odds of distal adenoma detection in univariable models (full dataset, descending colon: OR 1.43, 95%CI 1.31–1.56; SF: OR 1.66, 95%CI 1.47–1.88); however, this attenuated in multivariable models.

In complete-case univariable models, there were lower odds of distal adenoma detection with increasing pain (severe compared to none: OR 0.69, 95%CI 0.51–0.95; Table 1) but this was not evident in the other models. In the full dataset, individuals whose FS screening occurred after their endoscopist’s first 500 examinations had increased odds of distal adenoma detection compared to those whose took place earlier (OR 1.32, 95%CI 1.20–1.45); multivariable models were not possible due to missing data.

### Advanced and/or multiple adenomas

There were 919 (4.8%) participants with multiple and/or advanced distal adenomas in the complete-case dataset (Supplementary Table 6). Age, sex, family history, bowel preparation quality, insertion and withdrawal time, segment reached, patient pain and the order of FS occurrence were similarly associated with the detection of advanced and/or multiple adenomas as of any distal adenoma.

### Distal CRC incidence

During a median follow-up of 17 years, 168 (0.5%) distal CRCs were diagnosed (Table 2). In the full dataset, females had a lower risk of distal CRC than males (HR 0.62, 95%CI 0.45–0.85) and those with a family history of CRC had a higher risk than those without (HR 1.65, 95%CI 1.09–2.50) (Table 2, Supplementary Fig. 1A-B); these effects attenuated in complete-case models.

Age, bowel preparation quality, segment of bowel reached, patient-reported pain, and order of examination occurrence were not associated with distal CRC incidence (Table 2, Supplementary Fig. 1C–F). Although overall the associations for insertion and withdrawal times were not statistically significant, those in the top category of ≥ 4.00 min (versus < 2.00 min) had an increased risk of distal CRC (multivariable: HR 1.81, 95%CI 1.00–3.27; HR 1.93, 95%CI 1.14–3.24, respectively) (Table 2, Supplementary Fig. 1G-H).

Compared to those examined by high-detectors, individuals examined by low-detectors had an increased risk of distal CRC (multivariable: HR 4.71, 95%CI 2.65–8.38), as did those examined by intermediate-detectors in complete-case models only (multivariable: HR 2.16, 95%CI 1.22–3.81) (Table 2, Supplementary Fig. 1I).

Excluding participants with multiple FS examinations (*n* = 1,810) did not materially alter the results for distal adenoma detection or long-term colorectal cancer incidence in any of the models.

## Discussion

We investigated factors that could improve the quality of FS examinations, increase adenoma detection, and reduce CRC incidence. We found that multiple variables were associated with adenoma detection, including patient age, sex, family history of CRC, bowel preparation quality, insertion time and withdrawal time. For long-term outcomes, patients who were examined by endoscopists with higher ADRs had a lower risk of distal CRC incidence.

Individuals with a family history of CRC or its precursor lesions are at increased risk of CRC compared to those without [24]; similarly, we found a positive association between family history of CRC and distal adenoma detection at FS screening [25]. Participants provided family history information on the pre-screening questionnaire, which the endoscopist may have accessed, potentially motivating them to conduct a more thorough FS examination.

Bowel preparation plays a crucial role in the quality and completeness of endoscopic examinations, with higher levels of cleanliness associated with optimum views of the colon [26] and improved ADRs [23]. Compared to having ‘excellent’ bowel preparation, we found lower odds of adenoma detection among those having ‘good’ or ‘adequate’ and increased odds among those having ‘poor’. Among participants with poor bowel preparation at first FS, those who had adenomas detected that triggered referral to colonoscopy would not have had a repeat FS to improve the bowel preparation quality; however, those without high-risk adenomas detected would have undergone a repeat FS, likely improving the bowel preparation quality. This could contribute to poor bowel preparation being positively associated with adenoma detection. We were unable to examine poor bowel preparation quality and distal CRC incidence due to a lack of cases, attributed to the fact that we only included complete examinations.

In contrast to previous findings, which either reported no correlation between adenoma detection and longer insertion time [27] or decreased adenoma detection with longer insertion times [28, 29], we found that longer insertion time was associated with greater adenoma detection. Within the UKFSST, endoscopists were to remove polyps ≤ 5 mm during insertion to avoid difficulties relocating them on withdrawal, remove polyps 6-9 mm during withdrawal, and leave polyps ≥ 10 mm for removal at colonoscopy. Therefore, longer insertion times in this study could be associated with the presence of numerous polyps ≤ 5 mm requiring resection and/or very large adenomas needing endoscopic assessment/photo-documentation. We also found increased odds of detecting multiple and/or advanced adenomas in those with longer insertion times. In our study there was no fixed endpoint for FS examinations and further reach of the sigmoidoscope during an examination would naturally lead to longer insertion and withdrawal times and a higher chance of adenoma detection. However, in multivariable models we adjusted for segment reached, insertion time and withdrawal time. Quality of bowel preparation has been associated with longer insertion times [30, 31]. Advancing a sigmoidoscope through a bowel with poorer preparation requires more cleaning to obtain good views of the mucosa, potentially increasing insertion times. However, in our multivariable model including both insertion time and bowel preparation quality, the association between insertion time and adenoma detection remained.

Higher quality withdrawal techniques are associated with fewer missed adenomas. Colonoscopists with lower miss rates for adenomas had longer examination times compared to those with higher miss rates [19]. We found that longer withdrawal times were associated with increased adenoma detection, which is unsurprising due to the time taken to remove lesions < 10 mm during FS. Although larger lesions would not have been removed during FS, they would likely increase the examination time. For distal CRC incidence, only the longest withdrawal time category was associated with increased risk; this can likely be attributed to patients with long withdrawal times having more advanced pathology and inherently being at higher risk rather than reflecting the quality of the endoscopist’s withdrawal. There is no minimum recommended withdrawal time for FS, unlike for colonoscopy [20]. A previous study suggested a FS withdrawal time of at least 3.25 min from the SF and, to maximise ADRs, specified an aim of 3.5–4.0 minutes [14]; although this has not been validated, our data supports this recommendation.

FS with a 60 cm maximum scope insertion distance can reach the SF and sometimes beyond [32]. In our study, the majority (78%) of examinations were judged by the endoscopist to have reached at least the descending colon with 15% reaching at least the SF. It is important that the sigmoidoscope reaches as high as comfortably possible to maximise the mucosa examined, increasing the effectiveness of the examination [33, 34]. In our univariable analyses, the chance of detecting an adenoma was greater when at least the descending colon was reached, although this effect attenuated in multivariable models. Previously, inadequate examinations (e.g., insertion of the scope < 50 cm) were associated with female sex and advancing age, with the majority of incomplete examinations due to patient discomfort [34]. In agreement with this, we found decreased odds of reaching the SF for females, those who reported more pain, and those with poorer bowel preparation quality among those with negative examinations [14]. However, we found no clear association between patient-reported pain and adenoma detection or distal CRC incidence. Identifying factors that could reduce levels of pain could result in more complete examinations, lessening the chances of negative experiences that could compromise attendance at future examinations.

We found that adenomas were more likely to be detected at examinations conducted after an endoscopist’s first 500 examinations, suggesting a learning effect, consistent with previous analyses [15]; although we cannot be certain that participants examined within an endoscopist’s first 500 examinations had adenomas missed at baseline. It has been reported that for each 1% increase in the ADR, there is an associated 3% decreased risk of post-colonoscopy CRC [13] and that greater long-term protection from CRC is observed when FS screening is conducted by endoscopists with higher ADRs [35]. We found an almost five-fold increase in distal CRC incidence for individuals screened by low-detectors compared to those screened by high-detectors; this suggests an ADR of 15%, observed among the high-detectors, should be considered as a minimal standard. Other factors could account for differences in ADR, including variations in equipment, screening protocols or endoscopists’ prior experience; these factors were controlled for in the study design/analysis, which lends more weight to the difference in ADRs reflecting real variability in endoscopist performance and consequent effects on CRC incidence [5].

Although seven variables were associated with adenoma detection, only endoscopist ADR group was associated with distal CRC, in addition to insertion and withdrawal times in the top categories only. These differences potentially demonstrate the importance of certain factors in adenoma detection but not necessarily cancer prevention, but differences in findings could be due to a lack of power for the distal CRC analyses.

Strengths of our study include the large, high-quality dataset with multiple KPI measures and long follow-up period. Participants were recruited throughout the UK, resulting in good generalisability of our findings. Complete endoscopic examinations are crucial as incomplete examinations are associated with higher numbers of interval cancers [36, 37]; we only included examinations classed by the endoscopist as complete. In addition, we only included examinations performed by the main endoscopist at each centre, which removed heterogeneity within centres introduced by multiple endoscopists. There were limitations, including missing data for insertion and withdrawal times, potential inaccuracy in classifying depth of insertion since imaging systems were not used and limited statistical power for distal CRC analyses. We were unable to exclude examination time used for polyp removal or endoscopic assessment/photo documentation of polyps, which may have contributed to the association between adenoma detection and longer insertion and withdrawal times. Additionally, since the trial screening was conducted there have been advances in the quality of endoscopic equipment and improvements in endoscopist training and monitoring; therefore, the number of adenomas detected today would likely be higher.

In conclusion, there is a lack of published data on KPIs and long-term CRC outcomes. Examining the impact of KPIs on adenoma detection and distal CRC incidence, we identified several variables associated with patient outcomes. Examinations with good or adequate bowel preparation quality had lower odds of adenoma detection, and longer insertion and withdrawal times had increased odds of adenoma detection. Patients examined by endoscopists with high ADRs had the lowest risk of distal CRC. We suggest an ADR of 15% should be set as a minimal standard. The importance of the detection and removal of adenomas cannot be understated; early detection of abnormalities is key in providing long-term protection against CRC. It is vital that each endoscopic procedure is conducted to the highest standard, so all patients receive the optimum benefit that screening can offer.

Parts of the reported results have been presented as a poster presentation [38].

### Supplementary Information

**Additional file 1: Supplementary Table 1.** Reasons for repeat flexible sigmoidoscopy. **Supplementary Table 2.** Protocol guidelines for the categorisation of bowel preparation quality. **Supplementary Table 3.** Endoscopist variables by endoscopist for negative examinations^*^. **Supplementary Table 4.** Patient-reported pain and insertion time by extent of examination in negative examinations^*^. **Supplementary Table 5.** Reaching the splenic flexure in negative examinations^*^ by age, sex, bowel preparation quality, and pain. **Supplementary Table 6.** Detection of multiple and/or advanced distal adenomas by patient factors and endoscopist variables. **Supplementary Figure 1.** Cumulative distal colorectal cancer incidence in all eligible participants by patient factors and endoscopist variables (the full dataset).

## Data Availability

The data used in this current study is not available as it uses individual-level identifiable data, which is confidential. Requests regarding data should be directed to the corresponding author.

## Abbreviations

AC: Ascending colon
ADR: Adenoma detection rate
CI: Confidence interval
CM: Caecum
CRC: Colorectal cancer
DC: Descending colon
FS: Flexible sigmoidoscopy
HF: Hepatic flexure
HR: Hazard ratio
ICD-10: International Classification of Diseases 10^th^ revision
ISRCTN: International Standard Randomised Controlled Trial Number
IQR: Interquartile range
KPIs: Key performance indicators
Mins: Minutes
NHS: National Health Service
OR: Odds ratio
PIAG: Patient Information Advisory Group
RM: Rectum
RS: Rectosigmoid
SC: Sigmoid colon
SD: Sigmoid descending
SF: Splenic flexure
TC: Transverse colon
TI: Terminal ileum
UKFSST: UK Flexible Sigmoidoscopy Screening Trial
