# Supplementary material for: The impact of endoscopist performance and patient factors on distal adenoma detection and colorectal cancer incidence
Source: BMC Gastroenterol. 2024 Jan 23;24:44. doi: 10.1186/s12876-024-03125-x (PMC10804571; doi:10.1186/s12876-024-03125-x)
Supplement: Supplementary file 1 — Additional file 1: Supplementary Table 1. Reasons for repeat flexible sigmoidoscopy. Supplementary Table 2. Protocol guidelines for the categorisation of bowel preparation quality. Supplementary Table 3. Endoscopist variables by endoscopist for negative examinations*. Supplementary Table 4. Patient-reported pain and insertion time by extent of examination in negative examinations*. Supplementary Table 5. Reaching the splenic flexure in negative examinations* by age, sex, bowel preparation quality, and pain. Supplementary Table 6. Detection of multiple and/or advanced distal adenomas by patient factors and endoscopist variables. Supplementary Figure 1. Cumulative distal colorectal cancer incidence in all eligible participants by patient factors and endoscopist variables (the full dataset). [file 12876_2024_3125_MOESM1_ESM.docx]

**Supplementary Appendix**

**Supplementary Table 1.** Reasons for repeat flexible sigmoidoscopy.

| **Reason for repeat flexible sigmoidoscopy** | **Number of repeat examinations, n (%)^*†^** |
| --- | --- |
| Poor bowel preparation | 1 693 (89.2) |
| Warfarin/aspirin | 72 (3.8) |
| Pain | 32 (1.7) |
| To find polyps seen previously | 11 (0.6) |
| Check excision site | 7 (0.4) |
| Difficult polypectomy | 5 (0.3) |
| Bleeding | 3 (0.2) |
| Fainting/vasovagal | 2 (0.1) |
| Technical problem with equipment | 2 (0.1) |
| Safety of polypectomy | 1 (0.1) |
| Difficult to retain air | 1 (0.1) |
| Unknown | 69 (3.6) |
| **Total** | 1 898 (100) |

^*^1 810 individuals received multiple FS examinations (1 724 had two exams, 84 had three exams and two had four exams) and all repeat examinations are included.

^†^Because of rounding, percentages do not total to 100.0.

**Supplementary Table 2.** Protocol guidelines for the categorisation of bowel preparation quality.

| **Bowel preparation category** | **Definition** |
| --- | --- |
| Excellent | No stool or fluid present. |
| Good | Some residual liquid or stool present but removable by suction, allowing a completely reliable examination. |
| Adequate | Examination accomplished, but with difficulty due to residual stool. The reliability of the examination is suboptimal. |
| Poor | The examination could not be performed satisfactorily due to the presence of excess faeces. |

**Supplementary Table 3.** Endoscopist variables by endoscopist for negative examinations^*^.

| **Centre**^†^ | **Negative examinations,**  **n (%)** | **Bowel preparation quality**^‡^ | | | | **Insertion**  **time (mins)**^‡^ | **Withdrawal**  **time (mins)**^‡^ | **Segment reached**^‡^ | | | | | **Patient pain**^‡^ | | | |
| --- | --- | --- | --- | --- | --- | --- | --- | --- | --- | --- | --- | --- | --- | --- | --- | --- |
|  |  | **Excellent**  **n (%)** | **Good**  **n (%)** | **Adequate**  **n (%)** | **Poor**  **n (%)** | **Median (IQR)** | **Median (IQR)** | **RM/RS/**  **SC**  **n (%)** | **SD**  **n (%)** | **DC**  **n (%)** | **SF**  **n (%)** | **TC/HF/ AC/ CM/TI**  **n (%)** | **None**  **n (%)** | **Mild**  **n (%)** | **Quite a lot**  **n (%)** | **Severe**  **n (%)** |
| **1** | 2 024 (8.5) | 811 (40.2) | 1 083 (53.7) | 124 (6.1) | 0 (0) | 2.10 (1.63-2.83) | 1.25 (0.98-1.58) | 17 (0.8) | 148 (7.3) | 1 125 (55.6) | 657 (32.5) | 77 (3.8) | 543 (27.4) | 1 047 (52.9) | 322 (16.3) | 68 (3.4) |
| **2** | 2 345 (9.8) | 1 321 (56.8) | 693 (29.8) | 247 (10.6) | 63 (2.7) | - | - | 19 (0.8) | 696 (29.8) | 1 340 (57.3) | 236 (10.1) | 46 (2.0) | 1 055 (45.7) | 1 138 (49.2) | 111 (4.8) | 7 (0.3) |
| **3** | 2 075 (8.7) | 955 (46.2) | 766 (37.0) | 344 (16.6) | 4 (0.2) | 2.58 (2.08-3.00) | 1.83 (1.42-2.08) | 3 (0.1) | 410 (19.9) | 1 588 (76.9) | 16 (0.8) | 47 (2.3) | 859 (42.8) | 979 (48.8) | 154 (7.7) | 15(0.7) |
| **4** | 1 721 (7.2) | 1 174 (68.2) | 326 (18.9) | 197 (11.4) | 24 (1.4) | 1.45 (1.03-2.07) | 0.88 (0.65-1.27) | 4 (0.2) | 451 (26.2) | 1 171 (68.0) | 62 (3.6) | 33 (1.9) | 808 (48.2) | 797 (47.6) | 68 (4.1) | 3 (0.2) |
| **5** | 1 801 (7.5) | 606 (33.9) | 663 (37.0) | 483 (27.0) | 38 (2.1) | 2.97 (2.13-3.93) | 2.38 (1.90-3.06) | 0 (0) | 417 (23.2) | 1 202 (67.0) | 148 (8.2) | 28 (1.6) | 380 (21.6) | 1 007 (57.2) | 327 (18.6) | 45 (2.6) |
| **6** | 1 887 (7.9) | 850 (45.3) | 638 (34.0) | 383 (20.4) | 4 (0.2) | 1.68 (1.30-2.22) | 1.02 (0.75-1.47) | 2 (0.1) | 154 (8.2) | 1 536 (81.9) | 52 (2.8) | 131 (7.0) | 255 (13.7) | 1 048 (56.5) | 486 (26.2) | 66 (3.6) |
| **7** | 1 793 (7.5) | 1 183 (66.1) | 352 (19.7) | 239 (13.4) | 15 (0.8) | 2.75 (2.12-3.65) | 1.00 (0.74-1.38) | 24 (1.3) | 470 (26.2) | 1 101 (61.4) | 68 (3.8) | 129 (7.2) | 319 (18.2) | 1 008 (57.4) | 374 (21.3) | 55 (3.1) |
| **8** | 2 094 (8.8) | 1 046 (50.3) | 594 (28.6) | 428 (20.6) | 10 (0.5) | 2.28 (1.67-3.12) | 1.95 (1.52-2.57) | 5 (0.2) | 134 (6.4) | 1 761 (84.1) | 108 (5.2) | 85 (4.1) | 410 (20.0) | 1 077 (52.6) | 477 (23.3) | 84 (4.1) |
| **9** | 1 627 (6.8) | 156 (9.6) | 974 (59.9) | 489 (30.1) | 6 (0.4) | 1.82 (1.30-2.57) | 1.45 (1.08-1.98) | 5 (0.3) | 563 (34.6) | 895 (55.0) | 80 (4.9) | 83 (5.1) | 476 (29.8) | 881 (55.1) | 214 (13.4) | 28 (1.8) |
| **10** | 1 692 (7.1) | 775 (45.9) | 433 (25.6) | 475 (28.1) | 6 (0.4) | 3.88 (2.92-5.50) | 1.68 (1.17-2.55) | 1 (0.1) | 530 (31.4) | 898 (53.2) | 106 (6.3) | 154 (9.1) | 306 (18.5) | 920 (55.7) | 368 (22.3) | 59 (3.6) |
| **11** | 1 481 (6.2) | 268 (18.2) | 660 (44.7) | 545 (36.9) | 3 (0.2) | 2.63 (2.03-3.53) | 1.92 (1.37-3.13) | 6 (0.4) | 462 (31.2) | 642 (43.3) | 193 (13.0) | 178 (12.0) | 365 (25.3) | 838 (58.1) | 229 (15.9) | 10 (0.7) |
| **12** | 1 845 (7.7) | 1 015 (55.1) | 496 (26.9) | 319 (17.3) | 11 (0.6) | 2.03 (1.53-2.70) | 1.27 (0.98-1.68) | 3 (0.2) | 587 (31.8) | 1 166 (63.2) | 71 (3.9) | 17 (0.9) | 319 (17.7) | 1 014 (56.2) | 433 (24.0) | 39 (2.2) |
| **13** | 1 546 (6.5) | 390 (25.2) | 404 (26.1) | 716 (46.3) | 36 (2.3) | 2.62 (1.82-3.73) | 2.02 (1.49-2.67) | 2 (0.1) | 486 (31.5) | 428 (27.7) | 529 (34.2) | 100 (6.5) | 512 (33.8) | 835 (55.2) | 150 (9.9) | 16 (1.1) |
| **Total** | **23 931 (100)** | **10 550 (44.3)** | **8 082 (33.9)** | **4 989 (20.9)** | **220 (0.9)** | **2.33 (1.65-3.25)** | **1.48(1.02-2.13)** | **91 (0.4)** | **5 508 (23.1)** | **14 853 (62.2)** | **2 326 (9.7)** | **1 108 (4.6)** | **6 607 (28.2)** | **12 589 (53.8)** | **3 713 (15.9)** | **495 (2.1)** |

Abbreviations: AC=ascending colon. CM=caecum. DC=descending colon. HF=hepatic flexure. HR=hazard ratio. IQR=Interquartile range. Mins=minutes. RM=rectum. RS=recto sigmoid. SC=sigmoid colon. SD=sigmoid descending. SF=splenic flexure. TC=transverse colon. TI=terminal ileum.

^*^Negative examinations are defined as no distractions present in the colorectum, including no cancer or polyps detected and no biopsies performed.

^†^Centre is ranked in ascending order by endoscopist ADR.

^‡^90 missing values on bowel preparation quality; 9 717 missing values on insertion time; 9 733 missing values on withdrawal time; 45 missing values on segment reached; 527 missing values on patient-reported pain.

**Supplementary Table 4.** Patient-reported pain and insertion time by extent of examination in negative examinations**^*^**.

|  | **Females**^†^ | | | **Males**^†^ | | | **All participants**^†^ | | |
| --- | --- | --- | --- | --- | --- | --- | --- | --- | --- |
| **Segment of the bowel reached** | **Number of examinations, n (%)** | **Quite a lot or severe patient pain**^‡^  **n (%)** | **Insertion time (mins)**^§^  **Median (IQR)** | **Number of examinations, n (%)** | **Quite a lot**  **or severe patient pain**^‡^  **n (%)** | **Insertion time (mins)**^§^  **Median (IQR)** | **Number of examination, n (%)** | **Quite a lot**  **or severe patient pain**^‡^  **n (%)** | **Insertion time**^§^  **Median (IQR)** |
| RM/RS/SC | 51 (0.4) | 16 (31.4) | 2.34 (1.52-3.86) | 40 (0.4) | 4 (10.0) | 1.42 (1.00-2.25) | 91 (0.4) | 20 (22.0) | 1.97 (1.42-3.43) |
| SD | 3 536 (29.3) | 1 002 (28.3) | 2.65 (1.85-3.75) | 1 844 (16.3) | 365 (19.8) | 2.43 (1.70-3.35) | 5 380 (23.0) | 1 367 (25.4) | 2.58 (1.80-3.62) |
| DC | 7 162 (59.4) | 1 491 (20.8) | 2.37 (1.68-3.25) | 7 370 (65.2) | 918 (12.5) | 2.08 (1.52-2.93) | 14 532 (62.2) | 2 409 (16.6) | 2.22 (1.58-3.07) |
| SF | 1 020 (8.5) | 158 (15.5) | 2.37 (1.75-3.45) | 1 255 (11.1) | 101 (8.0) | 2.14 (1.58-2.90) | 2 275 (9.7) | 259 (11.4) | 2.23 (1.67-3.15) |
| TC/HF/AC/CM/TI | 287 (2.4) | 66 (23.0) | 3.23 (2.33-4.55) | 795 (7.0) | 81 (10.2) | 2.52 (1.90-3.50) | 1 082 (4.6) | 147 (13.6) | 2.67 (1.93-3.82) |
| Total | 12 056 (100) | 2 733 (22.7) | 2.47 (1.75-3.45) | 11 304 (100) | 1 469 (13.0) | 2.17 (1.57-3.02) | 23 360 (100) | 4 202 (18.0) | 2.33 (1.65-3.25) |

Abbreviations: AC=ascending colon. CM=caecum. DC=descending colon. HF=hepatic flexure. IQR=Interquartile range. Mins=minutes. RM=rectum. RS=recto sigmoid. SC=sigmoid colon. SD=sigmoid descending. SF=splenic flexure. TC=transverse colon. TI=terminal ileum.

^*^Negative examinations are defined as no distractions present in the colorectum, including no cancer or polyps detected and no biopsies performed.

^†^Includes participants with data on segment of the bowel reached and patient pain (45 participants were missing data on segment reached and 527 participants on patient pain; these values are not mutually exclusive).

^‡^P-value from test for trend was <0.001.

^§^Includes participants with data on segment of the bowel reached, patient pain and insertion time (n=13 888; 7 100 females and 6 788 males).

**Supplementary Table 5.**  Reaching the splenic flexure in negative examinations^*^ by age, sex, bowel preparation quality, and pain.

|  | **All eligible participants: Full dataset analysis (n=23,886)**^†^ | | | | **Participants with complete data on all variables: Complete-case analysis (n=23,273)**^‡^ | | | | | |
| --- | --- | --- | --- | --- | --- | --- | --- | --- | --- | --- |
|  | **n (%)**^§^ | **Examinations to reach the SF**  **n (%)** | **Univariable**  **OR (95%CI)** | **p-value**^¶^ | **n (%)**^§^ | **Examinations**  **to reach the SF**  **n (%)** | **Univariable**  **OR (95%CI)** | **p-value**^¶^ | **Multivariable**  **OR (95%CI)**^**^ | **p-value**^¶^ |
| **Total** | **23 886 (100)** | 3 434 (14.4) |  |  | **23 273 (100)** | 3 349 (14.4) |  |  |  |  |
| **Age (IQR), years** | **60.3 (57.9-62.8)** | 3 434 (14.4) | 0.99 (0.98-1.00) | 0.06 | **60.3 (57.9-62.8)** | 3 349 (14.4) | 0.99 (0.98-1.00) | 0.07 | 0.99 (0.98-1.00) | 0.14 |
| **Sex** | **23 886 (100)** | 3 434 (14.4) |  | <0.001 | **23 273 (100)** | 3 349 (14.4) |  | <0.001 |  | <0.001 |
| Males | 11 558 (48.4) | 2 096 (18.1) | 1 |  | 11 258 (48.4) | 2 044 (18.2) | 1 |  | 1 |  |
| Females | 12 328 (51.6) | 1 338 (10.9) | 0.55 (0.51-0.59) |  | 12 015 (51.6) | 1 305 (10.9) | 0.55 (0.51-0.59) |  | 0.57 (0.53-0.62) |  |
| **Bowel preparation quality** | **23 797 (99.6)** |  |  | <0.001 | **23 273 (100)** |  |  | <0.001 |  | <0.001 |
| Excellent | 10 532 (44.3) | 1 611 (15.3) | 1 |  | 10 315 (44.3) | 1 575 (15.3) | 1 |  | 1 |  |
| Good | 8 069 (33.9) | 1 165 (14.4) | 0.93 (0.86-1.01) |  | 7 894 (33.9) | 1 142 (14.5) | 0.94 (0.86-1.02) |  | 0.93 (0.86-1.02) |  |
| Adequate | 4 978 (20.9) | 638 (12.8) | 0.81 (0.74-0.90) |  | 4 861 (20.9) | 623 (12.8) | 0.82 (0.74-0.90) |  | 0.79 (0.71-0.87) |  |
| Poor | 218 (0.9) | 10 (4.6) | 0.27 (0.14-0.50) |  | 203 (0.9) | 9 (4.4) | 0.26 (0.13-0.50) |  | 0.25 (0.13-0.50) |  |
| **Patient pain** | **23 360 (100)** | 3 357 (14.4) |  | <0.001 | **23 273 (100)** |  |  | <0.001 |  | <0.001 |
| None | 6 597 (28.2) | 1 111 (16.8) | 1 |  | 6 578 (28.3) | 1 109 (16.9) | 1 |  | 1 |  |
| Mild | 12 561 (53.8) | 1 840 (14.6) | 0.85 (0.78-0.92) |  | 12 508 (53.7) | 1 835 (14.7) | 0.85 (0.78-0.92) |  | 0.90 (0.83-0.98) |  |
| Quite a lot | 3 708 (15.9) | 366 (9.9) | 0.54 (0.48-0.61) |  | 3 694 (15.9) | 365 (9.9) | 0.54 (0.48-0.61) |  | 0.61 (0.54-0.69) |  |
| Severe | 494 (2.1) | 40 (8.1) | 0.44 (0.31-0.60) |  | 493 (2.1) | 40 (8.1) | 0.44 (0.31-0.61) |  | 0.53 (0.38-0.73) |  |

Abbreviations: CI=confidence interval. OR=odds ratio. SF=splenic flexure.

^*^Negative examinations are defined as no distractions present in the colorectum, including no cancer or polyps detected and no biopsies performed.

^†^Includes participants with data on segment reached (45 participants were missing data on segment reached).

^‡^89 missing values on bowel preparation quality; 526 missing values on patient-reported pain (these values are not mutually exclusive).

^§^All n and percentage except the entry for age, which is median and interquartile range.

^¶^P-values were calculated with the likelihood ratio test.

^**^Multivariable model includes age, sex, bowel preparation quality and patient-reported pain.

**Supplementary Table 6.** Detection of multiple and/or advanced distal adenomas by patient factors and endoscopist variables.

|  | **All eligible participants: Full dataset analysis (n=34 139)** | | | | **Participants with complete data on all variables: Complete-case analysis (n=19 333)^*^** | | | | | |
| --- | --- | --- | --- | --- | --- | --- | --- | --- | --- | --- |
|  | **n (%)**^†^ | **Participants with multiple and/or advanced adenomas detected at baseline**  **n (%)** | **Univariable**  **OR (95%CI)** | **p-value^‡^** | **n (%)**^†^ | **Participants with multiple and/or advanced adenomas detected at baseline**  **n (%)** | **Univariable**  **OR (95%CI)** | **p-value^‡^** | **Multivariable**  **OR (95%CI)**^§^ | **p-value^‡^** |
| **Total** | **34 139 (100)** | **1 588 (4.7)** |  |  | **19 333 (100)** | **919 (4.8)** |  |  |  |  |
| **Age (IQR), years** | **60.3 (57.9-62.8)** | **1 588 (4.7)** | 1.04 (1.02-1.06) | <0.001 | **60.5 (58.0-62.9)** | 919 (4.8) | 1.04 (1.02-1.07) | <0.001 | 1.04 (1.01-1.06) | 0.005 |
| **Sex** | **34 139 (100)** | **1 588 (4.7)** |  | <0.001 |  |  |  | <0.001 |  | <0.001 |
| Male | 18 127 (53.1) | 1 159 (6.4) | 1 |  | 10 315 (53.4) | 656 (6.4) | 1 |  | 1 |  |
| Female | 16 012 (46.9) | 429 (2.7) | 0.40 (0.36-0.45) |  | 9 018 (46.6) | 263 (2.9) | 0.44 (0.38-0.51) |  | 0.58 (0.49-0.68) |  |
| **Family history of CRC** | **32 356 (94.8)** | **1 519 (4.7)** |  | 0.017 |  |  |  | 0.023 |  | 0.025 |
| No | 28 663 (88.6) | 1 316 (4.6) | 1 |  | 17 123 (88.6) | 792 (4.6) | 1 |  | 1 |  |
| Yes | 3 693 (11.4) | 203 (5.5) | 1.21 (1.04-1.41) |  | 2 210 (11.4) | 127 (5.7) | 1.26 (1.04-1.52) |  | 1.27 (1.03-1.55) |  |
| **Centre** | **34 139 (100)** | **1 588 (4.7)** |  | <0.001 |  |  |  | <0.001 |  | 0.002 |
| 1 | 2 413 (7.1) | 72 (3.0) | 1 |  | 1 607 (8.3) | 39 (2.4) | 1 |  | 1 |  |
| 2^¶^ | 3 438 (10.1) | 125 (3.6) | 1.23 (0.91-1.65) |  | - | - | - |  | - |  |
| 3 | 2 674 (7.8) | 110 (4.1) | 1.39 (1.03-1.89) |  | 1 452 (7.5) | 43 (3.0) | 1.23 (0.79-1.90) |  | 1.02 (0.64-1.64) |  |
| 4 | 2 131 (6.2) | 77 (3.6) | 1.22 (0.88-1.69) |  | 1 452 (7.5) | 54 (3.7) | 1.55 (1.02-2.36) |  | 1.63 (1.03-2.57) |  |
| 5 | 2 466 (7.2) | 142 (5.8) | 1.99 (1.49-2.65) |  | 1 844 (9.5) | 98 (5.3) | 2.26 (1.55-3.29) |  | 1.01 (0.67-1.52) |  |
| 6 | 2 733 (8.0) | 90 (3.3) | 1.11 (0.81-1.52) |  | 1 579 (8.2) | 44 (2.8) | 1.15 (0.74-1.78) |  | 1.08 (0.67-1.72) |  |
| 7 | 2 516 (7.4) | 97 (3.9) | 1.30 (0.96-1.78) |  | 1 741 (9.0) | 71 (4.1) | 1.71 (1.15-2.54) |  | 1.24 (0.80-1.92) |  |
| 8 | 2 839 (8.3) | 124 (4.4) | 1.48 (1.10-2.00) |  | 1 486 (7.7) | 73 (4.9) | 2.08 (1.40-3.08) |  | 1.18 (0.77-1.81) |  |
| 9 | 2 493 (7.3) | 127 (5.1) | 1.75 (1.30-2.34) |  | 1 617 (8.4) | 81 (5.0) | 2.12 (1.44-3.13) |  | 1.69 (1.11-2.58) |  |
| 10 | 2 532 (7.4) | 144 (5.7) | 1.96 (1.47-2.62) |  | 1 578 (8.2) | 84 (5.3) | 2.26 (1.54-3.33) |  | 0.94 (0.61-1.44) |  |
| 11 | 2 324 (6.8) | 131 (5.6) | 1.94 (1.45-2.60) |  | 1 202 (6.2) | 77 (6.4) | 2.75 (1.86-4.08) |  | 1.21 (0.79-1.85) |  |
| 12 | 2 799 (8.2) | 143 (5.1) | 1.75 (1.31-2.34) |  | 2 214 (11.5) | 122 (5.5) | 2.34 (1.63-3.38) |  | 1.31 (0.88-1.96) |  |
| 13 | 2 781 (8.1) | 206 (7.4) | 2.60 (1.98-3.42) |  | 1 561 (8.1) | 133 (8.5) | 3.74 (2.60-5.39) |  | 1.71 (1.15-2.54) |  |
| **Bowel preparation quality** | **33 609 (98.4)** | 1 515 (4.5) |  | 0.68 |  |  |  | 0.004 |  | <0.001 |
| Excellent | 14 573 (43.4) | 663 (4.5) | 1 |  | 7 819 (40.4) | 363 (4.6) | 1 |  | 1 |  |
| Good | 11 692 (34.8) | 520 (4.4) | 0.98 (0.87-1.10) |  | 6 922 (35.8) | 321 (4.6) | 1.00 (0.86-1.16) |  | 0.72 (0.60-0.86) |  |
| Adequate | 7 060 (21.0) | 315 (4.5) | 0.98 (0.85-1.12) |  | 4 553 (23.6) | 227 (5.0) | 1.08 (0.91-1.28) |  | 0.55 (0.45-0.67) |  |
| Poor | 284 (0.8) | 17 (6.0) | 1.34 (0.81-2.19) |  | 39 (0.2) | 8 (20.5) | 5.30 (2.42-11.61) |  | 3.53 (1.45-8.56) |  |
| **Insertion time** | **20 371 (59.7)** | **1 004 (4.9)** |  | <0.001 |  |  |  | <0.001 |  | <0.001 |
| <2.00 mins | 7 357 (36.1) | 281 (3.8) | 1 |  | 7 014 (36.3) | 253 (3.6) | 1 |  | 1 |  |
| 2.00-2.59 mins | 6 198 (30.4) | 304 (4.9) | 1.30 (1.10-1.53) |  | 5 894 (30.5) | 282(4.8) | 1.34 (1.13-1.60) |  | 1.32 (1.09-1.59) |  |
| 3.00-3.59 mins | 3 412 (16.7) | 184 (5.4) | 1.44 (1.19-1.74) |  | 3 221 (16.7) | 164 (5.1) | 1.43 (1.17-1.75) |  | 1.40 (1.12-1.75) |  |
| $\geq$4.00 mins | 3 404 (16.7) | 235 (6.9) | 1.87 (1.56-2.23) |  | 3 204 (16.6) | 220 (6.9) | 1.97 (1.64-2.37) |  | 1.83 (1.47-2.28) |  |
| **Withdrawal time** | **20 326 (59.5)** | **998 (4.9)** |  | <0.001 |  |  |  | <0.001 |  | <0.001 |
| <2.00 mins | 10 625 (52.3) | 134 (1.3) | 1 |  | 10 204 (52.8) | 126 (1.2) | 1 |  | 1 |  |
| 2.00-2.59 mins | 3 672 (18.1) | 104 (2.8) | 2.28 (1.76-2.96) |  | 3 479 (18.0) | 93 (2.7) | 2.20 (1.68-2.88) |  | 2.23 (1.68-2.94) |  |
| 3.00-3.59 mins | 1 837 (9.0) | 82 (4.5) | 3.66 (2.77-4.84) |  | 1 740 (9.0) | 75 (4.3) | 3.60 (2.69-4.82) |  | 3.58 (2.66-4.82) |  |
| $\geq$4.00 mins | 4 192 (20.6) | 678 (16.2) | 15.11 (12.50-18.25) |  | 3 910 (20.2) | 625 (16.0) | 15.22 (12.52-18.50) |  | 14.31 (11.62-17.61) |  |
| **Segment reached** | **34 075 (99.8)** | 1 587 (4.7) |  | <0.001 |  |  |  | 0.001 |  | 0.98 |
| RM/RS/SC | 128 (0.4) | 5 (3.9) | 1.11 (0.45-2.73) |  | 53 (0.3) | 2 (3.8) | 0.98 (0.24-4.06) |  | 0.79 (0.18-3.43) |  |
| SD | 7 510 (22.0) | 266 (3.5) | 1 |  | 4 447 (23.0) | 171 (3.8) | 1 |  | 1 |  |
| DC | 21 327 (62.6) | 1 016 (4.8) | 1.36 (1.19-1.56) |  | 11 840 (61.2) | 566 (4.8) | 1.26 (1.05-1.49) |  | 1.01 (0.83-1.23) |  |
| SF | 3 462 (10.2) | 195 (5.6) | 1.63 (1.35-1.96) |  | 2 062 (10.7) | 124 (6.0) | 1.60 (1.26-2.03) |  | 0.95 (0.71-1.26) |  |
| TC/HF/AC/CM/TI | 1 648 (4.8) | 105 (6.4) | 1.85 (1.47-2.34) |  | 931 (4.8) | 56 (6.0) | 1.60 (1.17-2.18) |  | 0.97 (0.69-1.36) |  |
| **Patient pain** | **33 323 (97.6)** | 1 539 (4.6) |  | 0.09 |  |  |  | 0.009 |  | 0.41 |
| None | 9 563 (28.7) | 477 (5.0) | 1 |  | 4 937 (25.5) | 274 (5.5) | 1 |  | 1 |  |
| Mild | 17 859 (53.6) | 816 (4.6) | 0.91 (0.81-1.02) |  | 10 561 (54.6) | 490 (4.6) | 0.83 (0.71-0.96) |  | 0.88 (0.75-1.04) |  |
| Quite a lot | 5 224 (15.7) | 222 (4.2) | 0.85 (0.72-1.00) |  | 3 378 (17.5) | 138 (4.1) | 0.72 (0.59-0.89) |  | 0.84 (0.67-1.06) |  |
| Severe | 677 (2.0) | 24 (3.5) | 0.70 (0.46-1.06) |  | 457 (2.4) | 17 (3.7) | 0.66 (0.40-1.08) | - | 0.86 (0.51-1.47) |  |
| **FS occurrence**^**^ | **34 139 (100)** | **1 588 (4.7)** |  | 0.0001 |  |  |  | - |  | - |
| First group 500 | 5 410 (15.8) | 199 (3.7) | 1 |  | - | - | - |  | - |  |
| Later groups 500 | 28 729 (84.2) | 1 389 (4.8) | 1.33 (1.14-1.55) |  | - | - | - |  | - |  |

Abbreviations: AC=ascending colon. CI=confidence interval. CRC=colorectal cancer. CM=caecum. DC=descending colon. FS=flexible sigmoidoscopy. HF=hepatic flexure. Mins=minutes. OR=odds ratio. RM=rectum. RS=recto sigmoid. SC=sigmoid colon. SD=sigmoid descending. SF=splenic flexure. TC=transverse colon. TI=terminal ileum.

^*^1 783 missing values on family history of CRC; 530 missing values on bowel preparation quality; 13 768 missing values on insertion time; 13 813 missing values on withdrawal time; 64 missing values on segment reached; 816 missing values on patient-reported pain (these values are not mutually exclusive).

^†^All n and percentage except the entry for age, which is median and interquartile range.

^‡^P-values were calculated with the likelihood ratio test.

^§^Multivariable model includes age, sex, family history of CRC, centre, bowel preparation quality, insertion time, withdrawal time, segment reached and patient-reported pain.

^¶^Centre 2 was omitted from the complete-case analyses due to a lack of recorded information for insertion or withdrawal times, as this information was not required until partway through the trial at which time centre 2 had already completed recruitment.

^**^Order of occurrence of FS examination was omitted from the complete-case analyses due to a lack of recorded information for insertion and withdrawal times for the category ‘first group 500’; this information was not required until partway through the trial at which time each endoscopist had already completed 500 examinations.

**Supplementary Figure 1.**  Cumulative distal colorectal cancer incidence in all eligible participants by patient factors and endoscopist variables (the full dataset).

**A.**

**B.**

**C.**

**D**.

**E.**

**F.**

**G.**

**H.**

**I.**
